# Maxillary Sinus Augmentation Combining Bio-Oss with the Bone Marrow Aspirate Concentrate: A Histomorphometric Study in Humans

**DOI:** 10.1155/2015/121286

**Published:** 2015-10-12

**Authors:** Paulo José Pasquali, Marcelo Lucchesi Teixeira, Thiago Altro de Oliveira, Luis Guilherme Scavone de Macedo, Antonio Carlos Aloise, André Antonio Pelegrine

**Affiliations:** ^1^Department of Implant Dentistry, São Leopoldo Mandic Dental School, 13 José Rocha Junqueira Street, 13045-755 Campinas, Brazil; ^2^Department of Prosthodontics, São Leopoldo Mandic Dental School, 13 José Rocha Junqueira Street, 13045-755 Campinas, Brazil

## Abstract

*Purpose*. To investigate the regenerative results obtained with the association of bone marrow aspirate concentrate using the Bone Marrow Aspirate Concentrate (BMAC) method to a xenogeneic bone graft (Bio-Oss) in sinus floor elevation. *Materials and Methods*. Using a randomized controlled study design in eight consecutive patients (age of 55.4 ± 9.2 years), 16 sinus floor lift procedures were performed with Bio-Oss alone (control group, CG, *n* = 8) or combined with bone marrow aspirate concentrate obtained via the BMAC method (test group, TG, *n* = 8). Six months after the grafting procedures, bone biopsies were harvested during implant placement and were analyzed by histomorphometry. *Results*. Histomorphometric analysis revealed a significantly higher amount (*p* < 0.05) of vital mineralized tissue in TG when compared to the CG (55.15 ± 20.91% and 27.30 ± 5.55%, resp.). For nonvital mineralized tissue, TG presented a statistically higher level of Bio-Oss resorption (*p* < 0.05) when compared with the CG (6.32 ± 12.03% and 22.79 ± 9.60%, resp.). Both groups (TG and CG) showed no significantly different levels (*p* > 0.05) of nonmineralized tissue (38.53 ± 13.08% and 49.90 ± 7.64%, resp.). *Conclusion*. The use of bone marrow concentrate obtained by BMAC method increased bone formation in sinus lift procedures.

## 1. Introduction

Inadequate bone quantity in the posterior maxilla secondary to pneumatization of the maxillary sinus and/or postextraction alveolar ridge resorption can compromise dental implant placement, therefore requiring site grafting prior to implant placement using techniques such as maxillary sinus floor elevation. The ideal material for bone grafting should be biocompatible, induce no host rejection, present no risk of disease transmission, promote support for bone regeneration, and have mechanical stability from the outset, which should be maintained throughout the healing period [1].

The maxillary sinus floor elevation technique described by Tatum and published by Boyne and James in 1980 [2] described autologous bone as the filling material for the sinus cavity, which is still regarded as the gold standard in bone reconstruction. If, on the one hand, autologous grafts present osteoconductive, osteoinductive, and osteogenic potential [3], on the other, they present some risks [4]. This has translated into a constant search for the development of biomaterials to substitute autologous bone grafts, for instance, xenografts [5, 6], homologous grafts [7, 8], and synthetic grafts [9, 10].

Bone substitute biomaterials lack cellularity, which has encouraged much research into the field of tissue engineering in order to combine autologous osteogenic cells with osteoconductive materials. Consequently, the bone marrow is currently the most explored source of autologous cells, [11] since it contains a number of bone regenerating cells, such as undifferentiated mesenchymal cells that can differentiate into osteoblasts [12, 13]. They also present an angiogenic potential due to the production and release of vascular endothelium growth factor [14], which is highly desirable for graft integration [15].

Osteoconductive graft enrichment in reconstructive surgery for maxillary sinus floor elevation can be performed using cells from a bone marrow aspirate concentrate obtained by centrifugation [16–19]. This method is regarded as simple and safe because it is performed using autologous material immediately before surgery [20].

Therefore, the aim of this study was to histomorphometrically evaluate the use of a bone xenograft enriched with autologous bone marrow aspirate concentrate (BMAC) for maxillary sinus floor lifting.

## 2. Materials and Methods

This study was conducted in the outpatient clinic of the Department of Implant Dentistry of the São Leopoldo Mandic Dental School (Campinas, SP, Brazil), upon approval by the research ethics committee (694.065/2014) and free and informed consent forms for all the patients.

The inclusion criteria involved completely edentulous patients needing implants in the posterior maxillary region with no more than 4 mm of remaining alveolar ridge, with need for maxillary sinus floor augmentation. The patients also committed to returning for follow-up appointments and to maintaining adequate oral hygiene. Patients were excluded if they had a history of neoplastic disease treated with radiotherapy or chemotherapy, if they were pregnant or breastfeeding, if they were receiving treatment or were affected by an illness that could have an effect on bone homeostasis, allergy to any of the materials used, and sinus pathologies, or if they were smokers.

**Figure 1:**
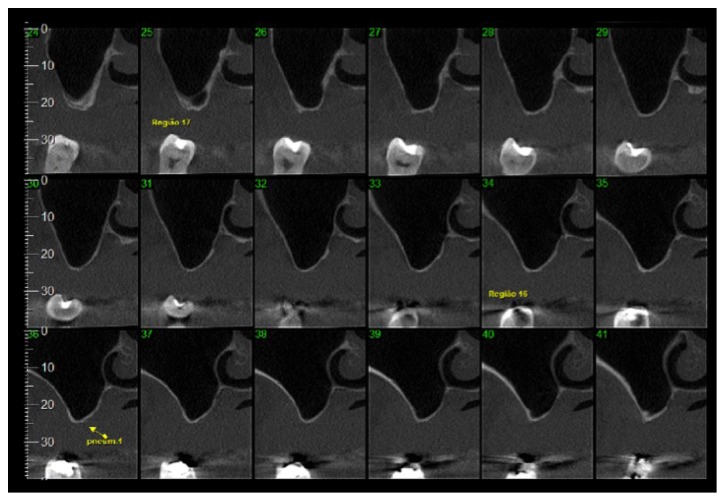
Cone-beam computed tomography scans of the posterior maxillary region.

Cone-beam computed tomography scans of the posterior maxillary region were obtained to measure the height of the posterior maxillary bone and the size of the maxillary sinus. The CT scans were also used to evaluate possible sinus pathologies (Figure 1).

Eight patients with a mean age of 55.4 ± 9.2 years were included in this study. They comprised sixteen atrophic maxillary sinuses to be grafted prior to implant placement. The patients were randomly divided using online-based software available at http://www.randomization.com/ into two groups according to the material used, control group (CG) (*n* = 8) with Bio-Oss only and test group (TG) (*n* = 8) with Bio-Oss combined with bone marrow concentrate obtained by the BMAC method, and each patient had the same graft material placed in each sinus. Following the principles of the guided bone regeneration technique, collagen membranes were placed over the bone window for all sinus floor augmentation procedures in both groups.

All patients were dentally rehabilitated using osseointegrated implants and fixed prostheses at the end of the study.

### 2.1. BMAC Method

**Figure 2:**
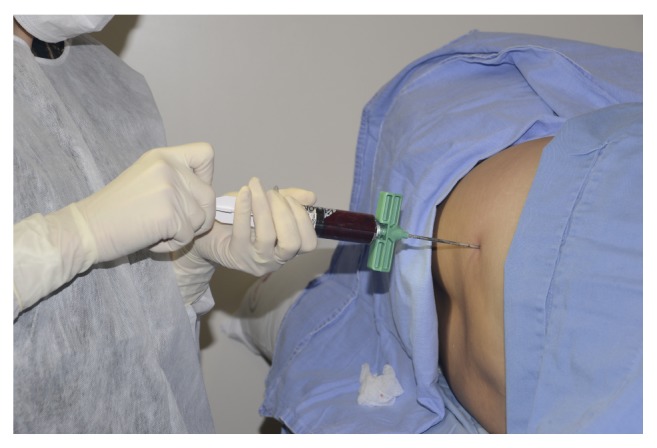
Bone marrow was collected from all the patients by aspiration through a puncture 2 cm laterocaudally from the superior posterior iliac crest.

According to the instructions by the manufacturer, bone marrow was harvested and processed directly in the operating room using the BMAC system (Bone Marrow Procedure Pack, Harvest Technologies, Plymouth, MA, USA). Briefly, in an outpatient setting and using local anesthesia (2% xylocaine without a vasoconstrictor), 30 mL of bone marrow was collected from all the patients by aspiration through a puncture 2 cm laterocaudally from the superior posterior iliac crest, using a bone marrow needle (included in the pack), with 30 mL syringes previously heparinized (1 mL of 5.000 U/mL heparin) (Figure 2).

**Figure 3:**
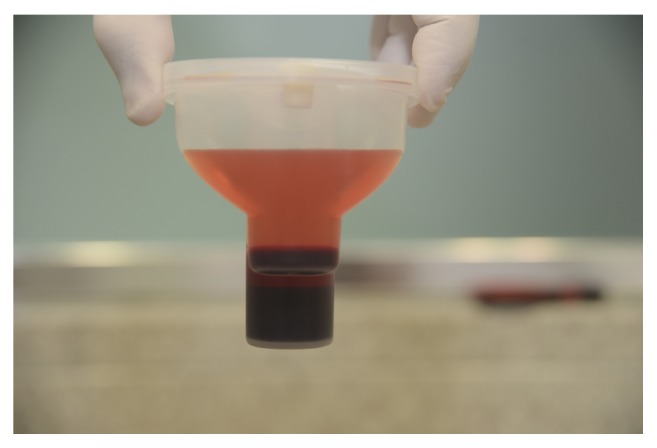
After centrifugation for 14 minutes, two phases were obtained within the container: (1) the supernatant plasma above and (2) precipitated bone marrow cells concentrate below.

The syringe containing 30 mL of bone marrow was connected to a filter bag, to which 8 mL of ACD-A anticoagulant was added. After appropriate homogenization, new syringe and needle were connected and the filtered 30 mL was removed. The bone marrow aspirate was then transferred into specific process disposables, which were placed in a SmartPReP2 centrifuge. After centrifugation for 14 minutes, two phases were obtained within the container, that is, the supernatant plasma and the precipitated bone marrow cells concentrate (Figure 3). The plasma was removed using specific syringes provided in the kit and the cell concentrate was resuspended and approximately 4 mL was aspirated.

### 2.2. Surgical Procedure

**Figure 4:**
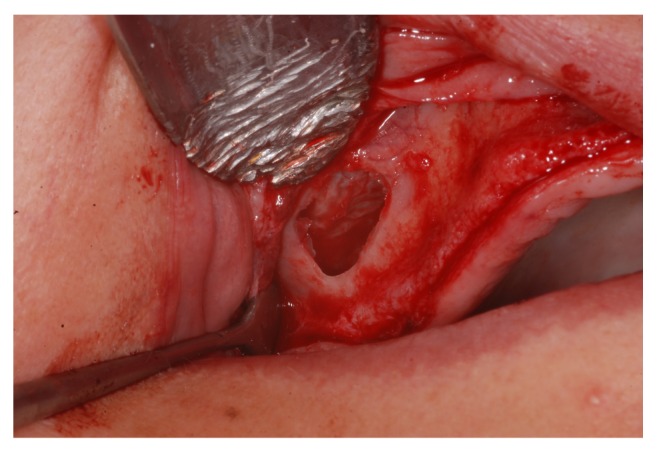
The Schneiderian membrane seen through the lateral window in the maxillary sinus.

**Figure 5:**
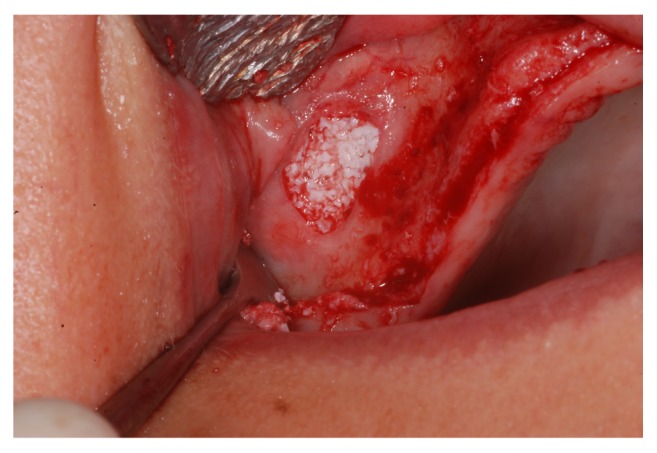
Maxillary sinus was grafted with xenogeneic bone from bovine hydroxyapatite.

**Figure 6:**
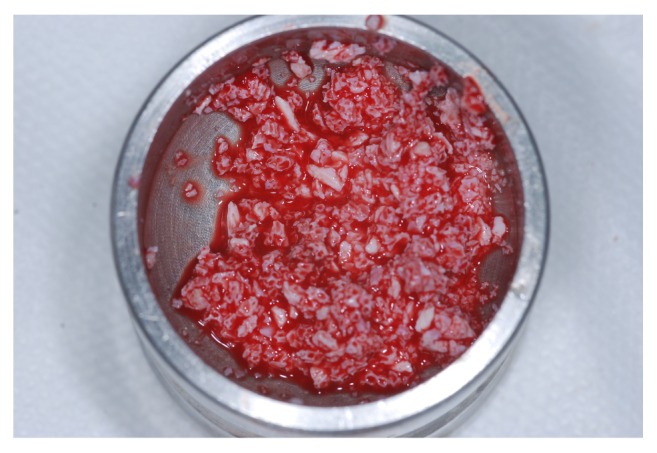
Xenograft combined with the bone marrow concentrate, immediately before being used.

A lateral window was prepared using number 3 PM spherical diamond bur (Medical Burs Ind. e Com. de Pontas e Brocas Cirúrgicas Ltda. Cotia, SP, Brazil) on the buccal aspect of the maxillary sinus. The Schneiderian membrane was carefully released (Figure 4) using specific curettes (Neodent, Curitiba, PR, Brazil) and the maxillary sinuses were grafted with xenogeneic bone from bovine hydroxyapatite, (1-2 mm Bio-Oss, Geistlich Biomaterials, Wolhusen, Switzerland) (Figure 5), either alone or combined with the bone marrow concentrate (Figure 6).

**Figure 7:**
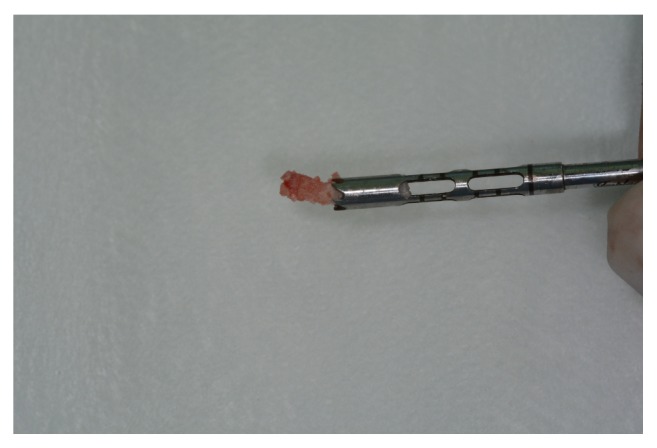
Bone specimens removed by a trephine bur.

**Figure 8:**
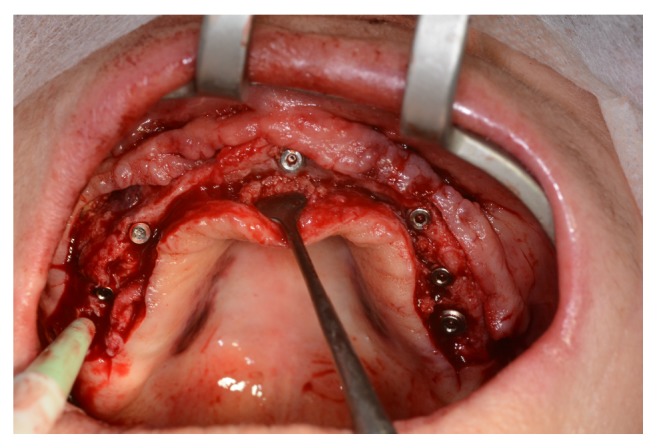
Dental implants (Blackfix, AS Technology, São José dos Campos, Brazil) immediately after they are placed.

A collagen membrane of porcine origin (Bio-Gide, Geistlich Biomaterials, Wolhusen, Switzerland) was used to cover the graft and the osteotomy of the lateral maxillary sinus wall, thus impeding migration of soft tissue to the graft region. After 6 months, 16 bone biopsies (one per sinus) were taken using a trephine bur (2.0 mm in diameter and 18 mm in length) (Figure 7). Immediately after that, the implants (Blackfix, AS Technology, São José dos Campos, Brazil) were placed (Figure 8). The bone biopsies were fixed in 4% formaldehyde solution (Merck, Darmstadt, Germany). The suture was made with ETHILON-Nylon 4-0 (Ethicon, MA, USA). After the surgery, each patient was prescribed Amoxicillin 500 mg (12/12 hours for 5 days). All patients were rehabilitated with metal-ceramic prosthesis over the implants six months after its installation.

### 2.3. Histological Analysis and Histomorphometric Measurements

The histological analysis was performed at the histopathology laboratory of the São Leopoldo Mandic Dental School (Campinas, SP, Brazil). The biopsies underwent decalcification in 10% EDTA for 36 hours and then processed following a conventional histological method for hard tissue. Subsequently, the samples were embedded in paraffin and 7-micrometer sections were cut. The entire area of the trephine biopsy above the native bone of the sinus was defined as region of interest and histomorphometrically evaluated.

The fragments were Masson's trichrome-stained and four different areas of each fragment were evaluated in the histology slides (upper left, lower left, upper right, and lower right), which were then averaged out.

**Figure 9:**
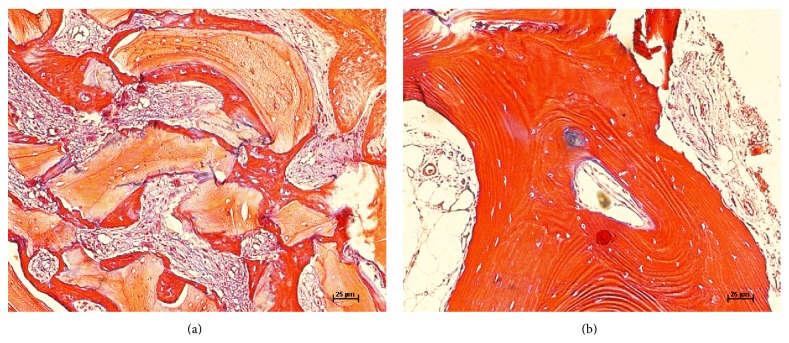
(a) Histological image of control group, stained by Masson's trichrome technique. Augmentation 20x. (b) Histological image of experimental group, stained by Masson's trichrome technique. Augmentation 20x.

Digital images were captured using a CCD digital camera (RT Cor., diagnostic instruments, Sterling Heights, MI, USA) coupled with an optical microscope (1.25x magnification). The digital images were merged to create a single image for each histological cut, using Adobe Photoshop Elements 2.0 software (Adobe Systems, San Jose, CA, USA) (Figures 9(a) and 9(b)).

Two previously trained examiners (AAP and ACA) blindly examined the images. Whenever disagreement occurred, the specimen was reevaluated and a consensus was reached. The examiners traced new bone formation on all the images using ImageJ Pro Plus 4.5 for Windows software (National Institute of Health, NIH, USA). The following parameters were considered for histomorphometry: (1) nonvital mineralized tissue (NVMT), (2) vital mineralized tissue (VMT), and (3) nonmineralized tissue (NMT). All the results were noted in square micrometers and, subsequently, stated as a percentage of the total area.

### 2.4. Statistical Analysis

For analysis of the nonvital mineralized tissue (NVMT), vital mineralized tissue (VMT), and nonmineralized tissue (NMT) parameters, values were stated as a percentage of the area evaluated. The nonparametric Mann-Whitney test was applied with correction using the Sidak-Bonferroni test for the statistical analysis. A value of *p* < 0.05 was considered significant.

## 3. Results

No membrane perforation was seen during the sinus lift procedures. At least two implants were placed in each previously grafted sinus and all of them were osseointegrated. Loading was applied after a 6-month healing period.

CG and the TG showed percentages of vital mineralized tissue (VMT) area of 27.30 ± 5.55% and 55.15 ± 20.91%, respectively. The same groups showed percentages of nonvital mineralized tissue (NVMT) area (represented by remaining Bio-Oss particles) of 22.79 ± 9.60% and 6.32 ± 12.03%, respectively. Finally, the percentages of nonmineralized tissue (NMT) area were 49.90 ± 7.64% and 38.53 ± 13.08%, respectively (Table 1).

## 4. Discussion

Surgery to augment the maxillary sinus is a well-documented method to generate adequate amount of bone for implant installation in the posterior maxilla [21–25], for which various types of bone grafts have been tested.

The autologous bone graft technique is considered the gold standard because of its osteoinductive, osteoconductive, and osteogenic characteristics. Nevertheless, it presents some drawbacks, especially regarding operative morbidity, namely, the need for two or more surgical sites in cases of greater amount of donor tissue, including extraoral sources. This raises the operative risk and surgical costs and generates postoperative discomfort, causing fewer patients to opt for this approach [4]. Consequently, a search for bone biomaterials that could replace the autologous bone has taken place. Nonetheless, these are not always graced with the advantages of osteogenesis and osteoinduction inherent of the autologous grafts [3].

Biomaterials that feature the physical, chemical, and mechanical characteristics of autologous bone have become increasingly desired, given the need for further use of the grafted area for the installation of dental implants. The literature reports on a xenogeneic bovine bone substitute, Bio-Oss, as a biomaterial with very similar characteristics to those of human bone, including osteoconduction [26–29]. Lyophilized xenogeneic bone tissue, or other bone substitute graft materials, lacks factors that promote osteogenesis and osteoinduction. In turn, this increases healing time compared to autologous bone, reaching a waiting period for implant placement ranging from six to eight months. This is greater than the time required for autologous grafts, which feature live cells and growth factors, therefore fulfilling their osteogenic and osteoinductive potentials [3]. Such properties reflect positively on the time required for bone healing, which may vary from four to six months [30].

Based on the aforementioned properties, methods to enhance bone substitutes combined with bone marrow cells have been investigated. Studies have described techniques for harvesting and applying fresh bone marrow [31–33] and isolating and expanding mesenchymal stem cells from the bone marrow [33–35] as well as concentrating the bone marrow cells [18, 33, 36, 37] in combination with a mineralized carrier. Despite these various methods, however, there is no consensus on the best alternative for bone remodeling in humans. Regarding the use of cell culture, mesenchymal stem cells must be carefully considered, because they usually require a waiting period of several weeks between harvesting, culture, and transplantation, thus risking contamination [38]. Therefore, in this study, preference was given to test a clinically plausible cell concentration method from a bone marrow aspirate concentrated by centrifugation within a fully closed system. The main reason for this choice was the versatility of the technique and the unlikelihood of contamination due to the fact that the system is closed. As stated by Sakai et al. [36], “to standardize the bone marrow transplantation for bone regeneration, a simple, safe, clean and cost-effective system is needed.”

In the present study, the waiting period of six months between grafting and reopening for implant placement was adopted, as it stands within the waiting period for autologous (4–6 months) and xenogeneic grafting (6–8 months). However, on the grounds that bone neoformation levels (vital mineralized tissue) in the test group were significantly higher (*p* = 0.002) than in the control group, 55.15 ± 20.91% and 27.30 ± 5.55%, respectively, one can speculate the possibility of early reopening when using the BMAC method. Sauerbier et al. (2010) achieved 19.9% of new bone after lifting maxillary sinuses with Bio-Oss associated with the BMAC method. The discrepancy between the results from both studies could probably be justified on the time delay between grafting and reopening surgery. In the study by Sauerbier et al. [37], a healing period of approximately 4.1 months was chosen, but in some cases, reopening occurred after just 3 months. The lack of time standardization for reopening combined with a precocious second intervention may have resulted in lower levels of bone tissue as stated by Sauerbier et al. [37] when compared to the present study, which standardized the reopening procedures at 6 months. Rickert et al. [18] compared the combination of Bio-Oss/BMAC method with Bio-Oss (70%) associated with autologous bone (30%) and detected no significant difference between the groups, further suggesting that the BMAC method improved the reconstructive standard. Nevertheless, the authors also opted for an early reopening time of approximately 3 months after grafting, which also hindered further comparisons between their data and those of the present study.

Regarding the levels of residual particles of Bio-Oss (NVMT), a significantly lower percentage (*p* = 0.006) was observed in the test group compared to the control, 6.32 ± 12.03% and 22.79 ± 9.60%, respectively. This can be hypothesized as an acceleration of the healing process in the test group, which corroborates the VMT results, since a higher rate of bone formation should translate into a higher biomaterial resorption. Regarding the levels of NMT, despite the lack of significant difference between the groups (*p* = 0.09), a decreasing trend in the amount of nonmineralized tissue in the test group was observed, which was, on average, 10% lower than in the control group.

There have been very few reports on the use of the biomaterials and techniques investigated in the present preliminary study. Therefore, further investigation is required to substantiate these results.

## 5. Conclusion

This study indicates that the clinical use of bone marrow aspirate concentrate obtained by the BMAC method associated with a xenograft for maxillary sinus elevation resulted in more adequate bone repair than the xenograft alone.

## Figures and Tables

**Table 1:** Statistical comparison of mean values (in percentage, %) between CG and TG.

Histomorphometric intergroup analysis			
Tissues	Groups		*p* value
	CG	TG	
NVMT	22.79 ± 9.60	6.32 ± 12.03	0.006
VMT	27.30 ± 5.55	55.15 ± 20.91	0.002
NMT	49.90 ± 7.64	38.53 ± 13.08	0.09

NVMT: nonvital mineralized tissue, VMT: vital mineralized tissue, NMT: nonmineralized tissue, CG: control group, and TG: test group.

Statistically significant *p* ≤ 0.05 (Mann-Whitney with correction by Sidak-Bonferroni test).
